# Measurements of Surface Impedance in MgB_2_ in DC Magnetic Fields: Insights in Flux-Flow Resistivity

**DOI:** 10.3390/ma16010205

**Published:** 2022-12-26

**Authors:** Andrea Alimenti, Kostiantyn Torokhtii, Pablo Vidal García, Enrico Silva, Mihai Alexandru Grigoroscuta, Petre Badica, Adrian Crisan, Nicola Pompeo

**Affiliations:** 1 Dipartimento di Ingegneria Industriale, Elettronica e Meccanica, Università degli Studi Roma Tre, Via Vito Volterra 62, 00146 Roma, Italy; 2 Istituto Nazionale di Fisica Nucleare–INFN, Sezione Roma Tre, Via della Vasca Navale 84, 00146 Roma, Italy; 3 National Institute of Materials Physics, 405A Atomistilor Street, 077125 Magurele, Romania

**Keywords:** MgB2, microwaves, microwave measurements, superconductors, surface impedance, vortex motion, dual band, flux-flow resistivity, pinning, upper critical field

## Abstract

We present the multifrequency measurements of the surface resistance of spark-plasma-sintered MgB${}_{2}$ performed through a dielectric loaded resonator operating at 16.5 and 26.7 GHz. By normally applying magnetic fields ≤1.2 T to the sample surface, we drove it in the mixed state. By means of data-rooted analysis, we found that the sample vortex dynamics could be fully described within a single-component approach. Pinning phenomena were present and characterized by a depinning frequency smaller than the measurement ones. The multiband nature of the superconductor emerged in the flux-flow resistivity, whose field dependence could be interpreted well within theoretical models. By exploiting them, the upper critical field was extracted in the low-temperature range, which exhibited a consistent temperature trend with the values obtained at the onset of the resistive transition near $T_{c}$, and was well in line with literature data on other polycrystalline samples.

## 1. Introduction

Magnesium diboride is a metal superconducting material that has sparked a lot of interest since the beginning from both a physical and a technological point of view [1]. Indeed, it has two superconducting bands of the BCS type [2], σ and π. The bands differ in the degree of anisotropy. Moreover, the π band is noticeably weaker than the other [1,3,4], so an intriguing physics emerges. Indeed, a complex interplay between two distinct superfluids and a variegate vortex phenomenology appear [5,6,7,8,9,10] and unusual vortex behaviors are predicted in different topological configurations [11,12,13]. From a technological point of view, its ease of production with low-cost materials and its medium-range critical temperature $T_{c}≃40$ K render it competitive in applications [14,15,16,17], particularly above 4.2 K [18,19]. Among the fabrication techniques, isostatic pressing, hot pressing, and spark-plasma sintering (SPS) [20,21,22] are particularly interesting.

Taking advantage of the specific unconventional features of SPS and its high flexibility by, for example, applying high heating and cooling rates, SPS routes are explored to increase its current-carrying capabilities. Often, the versatility of SPS in combination with the use of different additives results in the fabrication of high-quality MgB${}_{2}$ bulks with high compactness and effective pinning centers [23,24,25,26] or with certain mechanical properties such as machinability by chipping [16]. Recently, SPS of MgB${}_{2}$ also promoted the fabrication of bulks with a (001) texture and enhanced inplane superconducting properties [27].

SPS routes are explored to increase current-carrying capabilities by adding Te or cubic BN impurities, acting as effective pinning centers [23,24,28].

Given the relevance of these fabrication approaches, it is of interest to complement the DC studies on produced polycrystalline samples with microwave studies through surface impedance measurements given their capability to provide access to important fundamental properties [26]. Indeed, zero field measurements at microwaves on MgB${}_{2}$ were used to investigate the energy gap, the London penetration depth, the complex conductivity of samples, and the effect of disorder on both films and bulk polycrystals [29,30,31,32,33,34]. On the other hand, the microwave studies of vortex dynamics [35], predominantly performed on MgB${}_{2}$ epitaxial films and single crystals, have allowed for extracting the flux-flow resistivity $\rho_{ff}$ [36] and other pinning related quantities such as the depinning frequency [4,37] and the thermal creep factor [38,39].

In particular, flux-flow resistivity $\rho_{ff}$ is of particular interest given its role in setting the dissipation scale factor in the mixed state, and its sensitivity to the band structure and anisotropy of the superconducting material as well as to its disorder. Anomalous behavior consisting of a steep rise with the applied magnetic field followed by a down-curved trend, different from the standard Bardeen and Stephen behavior [40], has been observed since the early years [36] and interpreted in light of the peculiar two-band structure of MgB${}_{2}$ [41]. To assess whether this multiband signature is present in the flux-flow resistivity of sintered bulk polycrystalline samples, and to provide insights on the overall high-frequency vortex dynamics, in this work, we present multifrequency measurements on SPS-produced samples. The measurement technique, based on a dual-mode dielectric loaded resonator, is described in Section 2 and Section 3, the sample fabrication is summarized in Section 4.1, and the surface resistance and the resulting vortex parameters are reported in Section 4.2 and Section 4.3, respectively. Final comments are provided in Section 5.

## 2. Surface Resistance in the Mixed State

Surface impedance $Z_{s}$ is defined as the ratio of the tangential electric field and magnetic field of an electromagnetic (e.m.) wave impinging on a conductive flat surface. In the local limit and for bulk geometries, i.e., having thickness much larger than the EM wave penetration depth, it reads:

(1) $$Z_{s}=\sqrt{i\omega \mu_{0}\rho }\;,$$

where ω=2πν and ν are the angular frequency and frequency, respectively, and ρ is the resistivity of the material.

For single-band superconductors in the Meissner state, ρ is a complex quantity that can be expressed in terms of two-fluid conductivity [42]:

(2) $$\sigma_{2f}=\sigma_{1}-i\sigma_{2}=\sigma_{n}x_{n}-ix_{s}/\left(\omega \mu_{0}\lambda_{0}^{2}\right)\;,$$

where the second equality holds in the low-frequency limit ωτ≪1. Here, τ is the quasiparticle scattering time, $\sigma_{n}=1/\rho_{n}$ is the normal state conductivity, $\lambda_{0}$ the zero temperature penetration depth, and $x_{n}$ and $x_{s}$ are the normalized normal and superfluid fractions, respectively. At any reduced temperature $t=T/T_{c}$ (*T* and $T_{c}$ are the temperature and critical temperature, respectively) the normalization condition $x_{n}\left(t\right)+x_{s}\left(t\right)=1$ holds with the limits $x_{n}\left(0\right)=0$ and $x_{n}\left(1\right)=1$. For the dual-band superconductor MgB${}_{2}$, the Meissner state conductivity arises from the contribution of the π and σ bands, so that [4,31]

(3) $$\sigma_{2f}=\sigma_{2f,\pi }+\sigma_{2f,\sigma }\;.$$

By driving the superconductor in the mixed state with the application of a static magnetic field *H*, with $H_{c1}<H<H_{c2}$ ($H_{c1}$ and $H_{c2}$ are the lower and upper critical fields, respectively), vortices penetrate the material. Vortices are set in motion by the microwave oscillating currents and thus determine an additional contribution to the electrodynamic response represented by vortex motion resistivity $\rho_{vm}$. The latter combines with $\sigma_{2f}$ as follows [43]:

(4) $$\rho =\frac{\rho_{vm}+i/\sigma_{2}}{1+i\sigma_{1}/\sigma_{2}}\;.$$

In the microwave range (broadly speaking, 1–100 GHz), mean field–single vortex approaches can usually be followed, so that the vortex motion resistivity $\rho_{vm}$ for a single-band superconductor can be expressed as follows [35,43,44,45]:

(5) $$\rho_{vm}=\rho_{ff}\frac{1}{1-i\nu_{p}/\nu }\;,$$

where flux-flow resistivity $\rho_{ff}$ equal to $\rho_{n}B/B_{c2}$ within the Bardeen and Stephen (BS) model [40] sets the main scale for the dissipation due to vortex motion. Depinning frequency $\nu_{p}$ is the characteristic frequency that marks the crossover from the low-loss low-frequency regime, where $\rho_{vm}\to 0$, to a high-loss high-frequency regime, where $\rho_{vm}\to \rho_{ff}$. $\nu_{p}$ can be expressed as $\nu_{p}=k_{p}\rho_{ff}/\left(2\pi B\Phi_{0}\right)$, where pinning constant $k_{p}$ measures the intensity of the pinning recall forces acting on vortices when they are displaced from their equilibrium positions by the oscillating currents. In the above, creep effects were neglected: where the creep is finite, this approach would underestimate vortex parameters $\rho_{ff}$, $\nu_{p}$ and $k_{p}$ [45].

Multiband superconductors such as MgB${}_{2}$ exhibit a much more complex vortex phenomenology than the one captured by the above model due to the existence of multiple order parameters. Composite vortices are expected [5], and overlapping multicomponent cores yield attractive intervortex interaction [6,7,8]. Multicomponent vortex cores can be split into fractional vortices upon flux flow [10] and pinning action [46], potentially giving rise to two vortex systems with different flux flow resistivities and pinning effects. Further complexity in the field response of MgB${}_{2}$ arises due to pair-breaking effects, which occur with different field scales for the two bands, so that $x_{s,\pi }\left(t,B\right)$ differs from $x_{s,\sigma }\left(t,B\right)$. Indeed, experimental results suggest that a smaller π gap is suppressed earlier than a larger one [3,4].

Despite the intrinsic complexity of two-band superconductor MgB${}_{2}$, measurements of the surface impedance can provide very useful information when the analysis is performed in suitable field and temperature ranges, as described in the following.

## 3. Measurement Technique

Surface resistance $R_{s}=Re\left(Z_{s}\right)$ of MgB${}_{2}$ bulk disks was measured through the surface perturbation technique: the sample substitutes a base of a dielectric-loaded cylindrical resonator (DR) contributing to the overall electromagnetic losses that determine the resonator (unloaded) quality factor *Q*[47]. Thus, $R_{s}$ can be determined as follows:

(6) $$R_{s}\left(H,T\right)=G_{s}\frac{1}{Q(T,H)}-\mathrm{background}\;,$$

where $G_{s}$ is an analytically computed geometric factor or determined through finite-element simulations. The “background” term accounts for the losses occurring in the resonator itself (metal enclosure and dielectric rod). They can be separately evaluated by measuring the resonator quality factor without the inserted sample [47]. Quality factor *Q* is determined by inserting the resonator into a microwave line, to which it is coupled through two ports, and by exciting properly selected resonant modes. By using a vector network analyzer (VNA), the frequency-dependent scattering coefficients of the resonator both in transmission and reflection are measured around the resonant frequency of the selected modes. The calibration of the microwave line connecting the resonator to the VNA performing the measurements is taken into account together with the modeling of the contributions of the uncalibrated portions of the line within the cryostat [48,49,50]. The used resonator consists of a dual-tone [51] OFHC copper cylindrical resonator loaded with a single crystal sapphire (height 4.50 mm and diameter 7.13 mm), designed to operate with two distinct modes, TE${}_{011}$ and TE${}_{021}$. They have two different frequencies, $\nu_{1}=16.5$ GHz and $\nu_{2}=26.7$ GHz, thus enabling the dual-frequency measurements of the sample surface resistance. All details of the method, including the estimates of the uncertainties, can be found in [47,48,49,50,51].

## 4. Experimental Section

### 4.1. Sample Preparation

**Figure 1:**
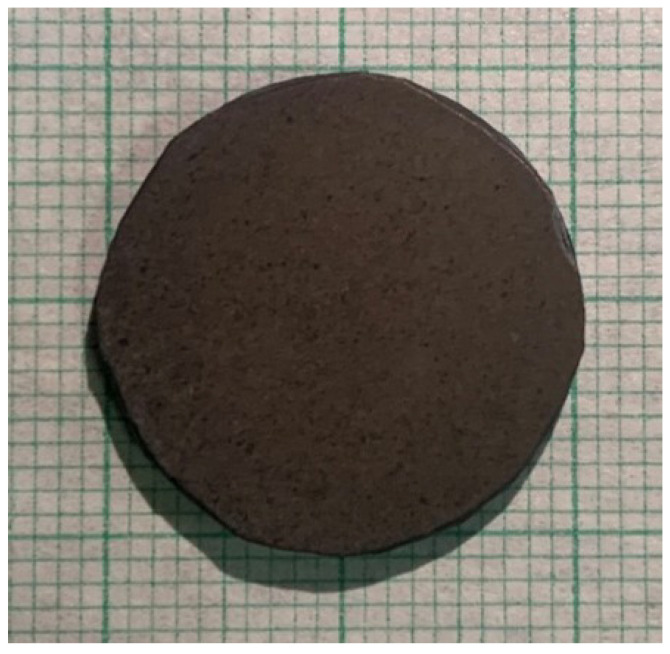
MgB${}_{2}$ disc fabricated with SPS used in microwave experiments.

Disk-shaped MgB${}_{2}$ bulk samples were fabricated by means of an ex-situ spark-plasma sintering technique (SPS). The raw powder of MgB${}_{2}$ supplied by Alfa Aesar (99.5% metal basis purity) was wrapped into graphite foil, loaded into a graphite die with punches, and sintered in vacuum (initial pressure of ∼30 Pa) at 1150 ${}^{\circ }$C for 3 min by using a FCT Systeme GmbH – HP D5 (Effelder-Rauenstein, Germany) furnace. The heating rate was ∼150 ${}^{\circ }$C/min, and the maximal uniaxial pressure applied on the sample during SPS was 95 MPa. After extracting the bulk from the die and cleaning the graphite from the surface with sandpaper, the final sintered MgB${}_{2}$ sample had a diameter of 20 mm and a thickness of 3.20 mm (Figure 1). The thickness was much larger than both the London penetration depth and the skin depth at the frequencies of interest. More details on sample preparation and characterization were presented in [23].

### 4.2. Surface Resistance

The MgB${}_{2}$ sample was measured in field cooling conditions by applying a static magnetic field $\mu_{0}H\leq 1.2T$, normal to the sample surface. The alternative approach, consisting in performing zero field cooling (ZFC) down to a desired *T*, thermalizing, and then measuring during a field sweep, was not feasible. Due to the ZFC condition, the bulk sample provided relatively high magnetization, resulting in a mechanical torque that prevented the good mechanical stability of the measurement cell. The need for the field cooling condition also prevents varying the orientation of the magnetic field for anisotropic studies [52], unless a time-consuming complete warming–cooling cycle is performed for each field intensity–field orientation pair.

**Figure 2:**
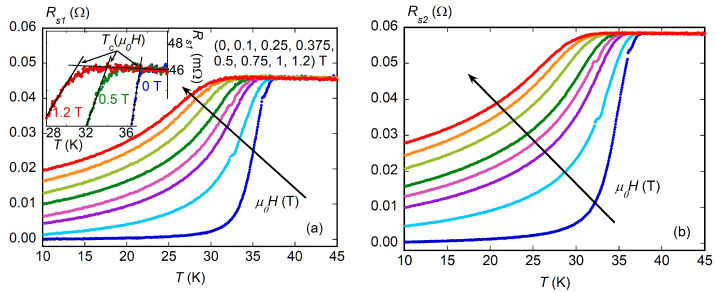
Surface resistance $R_{s}$ vs. *T* measured with different applied magnetic fields $\mu_{0}H$ at the frequencies of $\nu_{1}=16.5$ GHz in (**a**) and $\nu_{2}=26.7$ GHz in (**b**). In the inset of (**a**), the procedure used for the determination of the critical temperature $T_{c}$ at different applied fields is shown.

The measurement of the resonator *Q* factor at the two frequencies of $\nu_{1}=16.5$ GHz and $\nu_{2}=26.7$ GHz yielded two surface resistances, $R_{s1}$ and $R_{s2}$, respectively. Results are reported in Figure 2. By applying a finite field *H*, $R_{s}\left(T\right)$ increased, indicating that, as expected, vortex motion occurred, giving a dissipation whose main scale factor was flux-flow resistivity $\rho_{ff}\left(H\right)$; see Equation (5).

**Figure 3:**
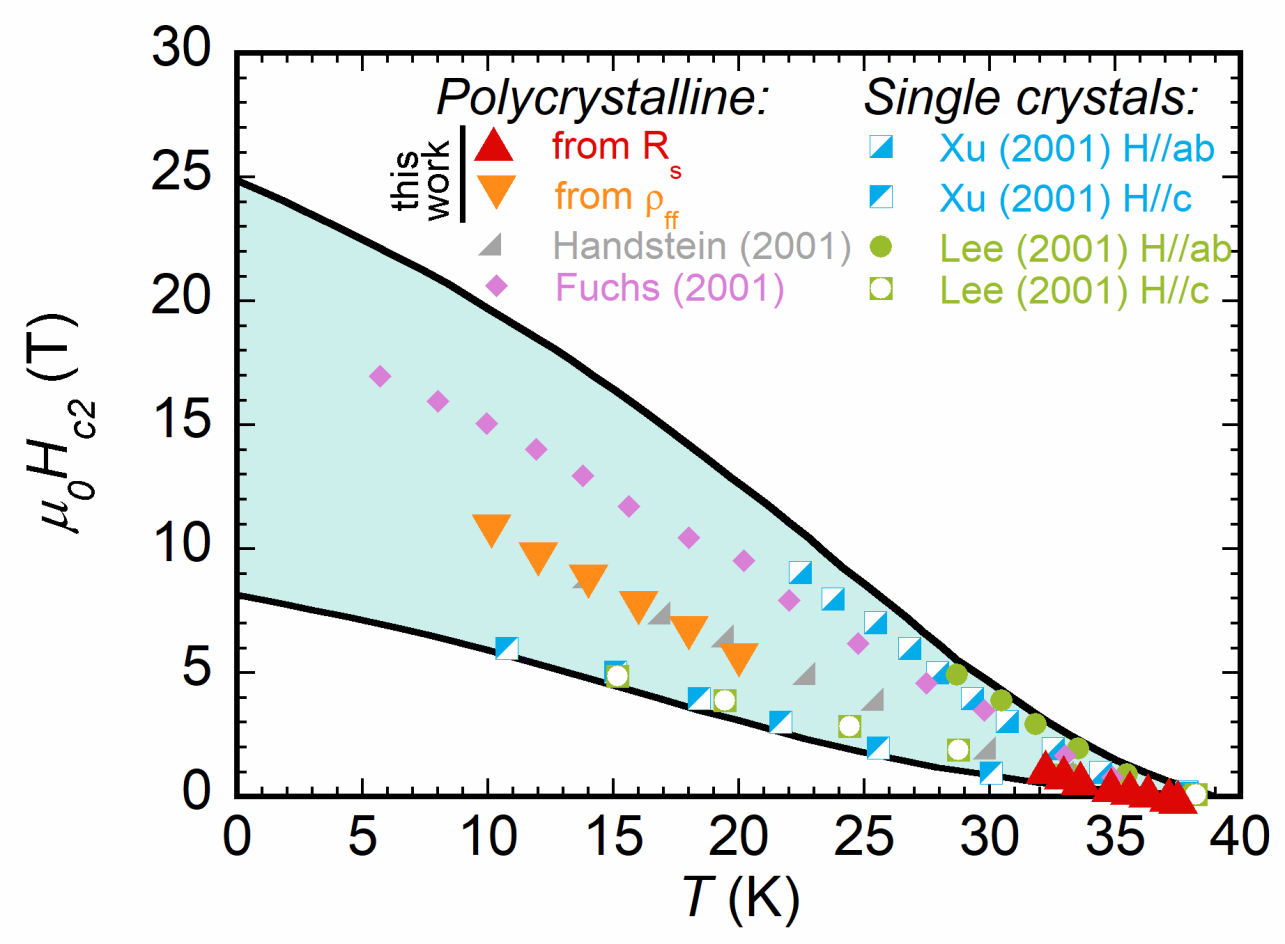
$H_{c2}$ as derived from the surface resistance data of Figure 2, compared to reported data in the literature as measured in polycrystalline samples [53,54] and in single crystals; in the latter case, with the applied magnetic field oriented both parallel to the *a*–*b* planes and the *c* axis. The continuous lines were the fit of anisotropic data, taken from [1]. The data obtained on the polycrystalline sample studied in this work were consistent with the values measured in other polycrystals and fell well between the two limits of $H_{c2}$ measured on oriented single crystals.

From zero field measurements, the sample critical temperature $T_{c}=37.5K$ could be obtained. Above $T_{c}$, all curves taken at different *H* coalesced, showing that normal state surface resistance $R_{n}$ was independent of the applied field and, since no slope was detected, independent from *T* in the measured temperature range. The saturation to $R_{n}$ at various fields allowed for directly evaluating $H_{c2}\left(T\right)$, according to the procedure sketched in the inset of Figure 2, whose values are plotted in Figure 3 with an extensive comparison with the literature (discussed later).

Since the sample was electromagnetically thick, that is, its thickness was much larger than both the skin depth and the London penetration depth, the conventional expression of

(7) $$R_{n}=\sqrt{\mu_{0}\omega \rho_{n}/2}\;,$$

allowed for the determination of normal state resistivity $\rho_{n}$. Within measurement uncertainties, we obtained the same $\rho_{n}=3.3\;\mu \Omega$cm from $R_{n}$ at frequencies $\nu_{1}$ and $\nu_{2}$, confirming that the sample was indeed in the bulk regime (no finite thickness effects that would bring changes to the relation between $R_{n}$ and $\rho_{n}$ with respect to Equation (7)).

**Figure 4:**
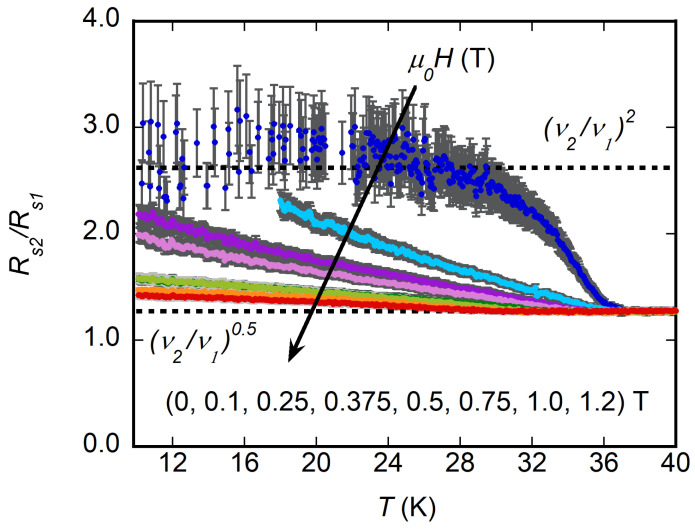
Ratio of the surface resistance measured at $\nu_{2}=26.7$ GHz and $\nu_{1}=16.5$ GHz, $r_{21}=R_{s2}/R_{s1}$ in MgB${}_{2}$ at different applied magnetic fields.

Further information can be drawn by considering the ratio of $r_{21}=R_{s2}/R_{s1}$ reported in Figure 4. We first focusd on the data obtained at H=0: at low temperature, $r_{21}≃2.7$, and in the normal state at $T>T_{c}$, $r_{21}≃1.27$. Above $T_{c}$, Equation (7) holds, yielding a theoretical ratio $R_{n}\left(\nu_{2}\right)/R_{n}\left(\nu_{1}\right)=\sqrt{\nu_{2}/\nu_{1}}=1.27$, in agreement with the measured $r_{21}$. At a low enough $T\ll T_{c}$, in Equation (2) one has $\sigma_{1}\ll \sigma_{2}$, and Equation (1) yields $R_{s}≃\omega^{2}\mu_{0}^{2}\lambda^{3}\sigma_{1}/2$ whence the theoretical ratio $R_{s}\left(\nu_{2}\right)/R_{s}\left(\nu_{1}\right)={\left(\nu_{2}/\nu_{1}\right)}^{2}=2.62$, in fair agreement with the experimental $r_{21}$, at low temperature. Thus, this preliminary scaling analysis at zero field confirms that the sample surface resistance was consistent with the model of bulk surface impedance within the local limit, enabling its use in the analysis.

The analysis of $r_{21}$ in a finite magnetic field provides useful information on the vortex pinning regime. Indeed, for zero pinning ($k_{p}=0$), Equation (5) immediately gives real vortex motion resistivity $\rho_{vm}\left(k_{p}=0\right)=\rho_{ff}$: with a negligible two-fluid contribution, which holds true far enough from the $H_{c2}\left(T\right)$ and $H_{c1}\left(T\right)$ lines; this would yield the same constant $r_{21}=\sqrt{\nu_{2}/\nu_{1}}$ as that in the normal state, in disagreement with the experimental data. The observed curves for $r_{21}\left(T\right)$ started at values well above $\sqrt{\nu_{2}/\nu_{1}}$ at low temperature, decreased with increasing *T*, eventually approaching the constant value in the normal state at $T_{c}$. This behavior immediately indicated that pinning was present, with a decreasing relevance approaching $T_{c}$. Hence, the interpretation of $R_{s}\left(\nu ,B,T\right)$ requires a vortex model including pinning.

**Figure 5:**
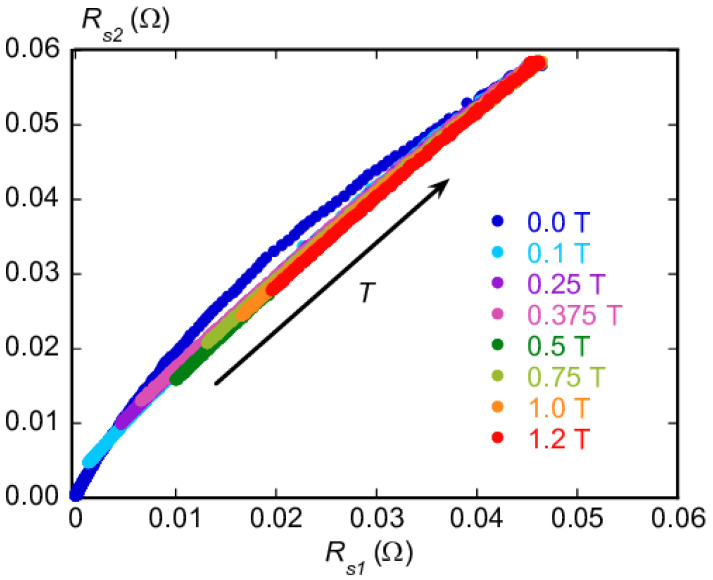
Surface resistance $R_{s}\left(\nu_{2}\right)$ vs. $R_{s}\left(\nu_{1}\right)$ with *T* and *B* as parameters. In particular, curves wre drawn by varying *T*, while different curves correspond to distinct *B* values.

As mentioned in Section 2, multigap superconductors such as MgB${}_{2}$ can exhibit a complex vortex phenomenology due to the existence of multiple order parameters. As a consequence, the model for the vortex motion resistivity could, in principle, be more complex than the single-component one discussed in Section 2. Thus, it is necessary to assess the modeling approach to be pursued. Insights can be gained by considering the parametric plot of $R_{s}\left(\nu_{2}\right)$ against $R_{s}\left(\nu_{1}\right)$, as reported in Figure 5. In this plot, temperature *T* was varied as an internal parameter.

The curves for finite fields $B=\mu_{0}H$ (London limit) superimposed quite well among themselves, especially at high $R_{s}$ values (that translates to higher temperatures). By contrast and for comparison, the zero field curve stood evidently aside. The collapse of all the curves taken in magnetic field points to a single field scale (at least not too close to the origin of the plot, i.e., low temperatures): looking at Equation (5), it is immediately apparent that two field scales appeared in the vortex motion resistivity if $\nu_{p}$ depended on *H*. In addition, two-band superconductivity might produce a more complex vortex motion response well. However, the scaling of Figure 5 points to an important result: single-component vortex motion resistivity, possibly with an effective vortex parameter capturing the contributions from the two bands, should be able the describe the experimental data. Indeed, mathematically, the scaling indicates that surface resistances $R_{s}\left(\nu_{1},B,T\right)$ and $R_{s}\left(\nu_{2},B,T\right)$ shared the same (B,T) dependence, that is, there was a common function f(B,T) such as $R_{s1}=R_{s}\left(\nu_{1},f\left(B,T\right)\right)$ and $R_{s2}=R_{s}\left(\nu_{2},f\left(B,T\right)\right)$. To determine function f(B,T), suitable expressions for $R_{s}$ could be worked out. In the framework in which a single component $\rho_{vm}$ is dominant with respect two-fluid conductivity $\sigma_{2f}$, $R_{s}\left(\nu \right)$ becomes:

(8) $$\begin{array}{cc} R_{s}≃ℜ\left(\sqrt{i\mu_{0}\omega \rho_{vm}}\right) & =ℜ\left(\sqrt{i\mu_{0}\omega \rho_{ff}\left(B,T\right)\frac{1}{1-ir(B,T)}}\right)=\\ & =\sqrt{\frac{\mu_{0}\omega \rho_{ff}\left(B,T\right)}{2}}\sqrt{\frac{\sqrt{1+r{\left(B,T\right)}^{2}}-r\left(B,T\right)}{1+r{\left(B,T\right)}^{2}}}\;, \end{array}$$

where for compactness’ sake, $r\left(B,T\right)=\nu_{p}\left(B,T\right)/\nu$ represents the normalized depinning frequency. To exploit more information from the scaling observed in Figure 5, we examined two different limits for $R_{s}$.

In limit $\nu_{p}\gg \nu_{1},\nu_{2}$, that is, r≫1 (known as Campbell regime), one has $R_{s}=\omega^{2}\sqrt{\mu_{0}\rho_{ff}\left(B,T\right)/\left(32\pi^{3}\nu_{p}^{3}\left(B,T\right)\right)}$. Hence, $f\left(B,T\right)=\rho_{ff}\left(B,T\right)/\nu_{p}^{3}\left(B,T\right)$; however, in this case, overall frequency dependence comes out, so that ratio $r_{21}$ should stay constant, as discussed previously, in disagreement with the data of Figure 4. Thus, this regime can be ruled out.

Another simple limit is $\nu_{p}\ll \nu_{1},\nu_{2}$ (r≪1), in which $R_{s}$ can be cast in the form of $R_{s}=\sqrt{\omega \mu_{0}\rho_{ff}\left(B,T\right)/2}\left(1-r\left(B,T\right)/2\right)$. It is easy to check that the scaling would occur with the single $f\left(B,T\right)=\rho_{ff}\left(B,T\right)$ only if (1−r(B,T)/2) yields negligible *B*-dependence, which happens if $\nu_{p}\left(B,T\right)$ is *B* independent or if *r* is sufficiently ≪1. In this case, the ratio $r_{21}=R_{s}\left(\nu_{2}\right)/R_{s}\left(\nu_{1}\right)$ should scale approximately as $\sqrt{\nu_{2}/\nu_{1}}$. According to Figure 4, apart from the lowest field measurements, $r_{21}\geq \sqrt{\nu_{2}/\nu_{1}}$ with a slight dependence on *B*. This residual field dependence excludes totally negligible pinning contribution (i.e., negates $\nu_{p}\ll \nu$), but the small magnitude of the residual field dependence confirmed that $\nu_{p}$ is indeed smaller than the measurement frequency.

From this whole scaling analysis, we can conclude that (i) a single-component vortex motion resistivity could be used in the interpretation of data, and (ii) pinning phenomena are present with a depinning frequency smaller than the measuring frequencies. We are now in the position to perform quantitative analysis that allows for extracting the actual vortex parameters.

### 4.3. Vortex Parameters

So far, we obtained some relevant qualitative information from the analysis of the data. In particular, we assessed the applicability of the physical models discussed in Section 2 to the data surface resistance data presented in Section 4.2. This is nontrivial in a multiband superconductor. Moreover, we determined that the measuring frequencies were above the depinning frequency. We now exploit the model to extract the vortex parameters from the data.

For each *H* and *T* data point, two experimental data were available, i.e., $R_{s}\left(\nu_{1}\right)$ and $R_{s}\left(\nu_{2}\right)$. The full model, including two-fluid conductivity, contains six independent parameters: real and imaginary parts of the two fluid conductivity for the two bands, and the two vortex parameters of interest: $\rho_{ff}$ and $\nu_{p}$ for the single component vortex resistivity. It is, thus, necessary to reduce the parameter space, as already described in [55] and briefly recalled here.

If we restrict the analysis to the low-temperature range 10K≤T≤20K and in the field range $0.5T\leq \mu_{0}H\leq B_{max}=1.2T$, the weaker π-band is almost completely suppressed [3,4], while the larger σ-band is well below the critical line $H_{c2}\left(T\right)$, being $B_{c2}\left(T=0\right)=$ 13 T to 20 T in bulk samples [1]. Hence, $\sigma_{2f,\pi }=\sigma_{n,\pi }$, but $\sigma_{n,\pi }\gg \sigma_{1,\sigma }$, so that $\sigma_{2f}≃\sigma_{n,\pi }-i\sigma_{2,\sigma }=\sigma_{n,\pi }-i/\left(\omega \mu_{0}\lambda^{2}\right)$. Real resistivity $\sigma_{n,\pi }$ can be derived from the measured $\rho_{n}=3.3\;\mu \Omega$cm (see Section 4.2), since $\rho_{n}={\left(\sigma_{n,\pi }+\sigma_{n,\sigma }\right)}^{-1}=\sigma_{n,\pi }^{-1}{\left(1+\sigma_{n,\sigma }/\sigma_{n,\pi }\right)}^{-1}$.

Ratio $\sigma_{n,\sigma }/\sigma_{n,\pi }={\left(\omega_{pl,\sigma }/\omega_{pl,\pi }\right)}^{2}\left(\Gamma_{\pi }/\Gamma_{\sigma }\right)$, where $\omega_{pl}$ and Γ are the plasma frequency and scattering rates, respectively, different for the two bands [31]. While ${\left(\omega_{pl,\sigma }/\omega_{pl,\pi }\right)}^{2}=0.494$ [56], the ratio of the scattering rates depends on the material disorder and varies among samples. A value $\sigma_{n,\sigma }/\sigma_{n,\pi }=0.5$ was derived by analyzing microwave vortex motion resistivity measurements in thin films with H‖c axis [4]; values 0.26 and 0.5 were used in the fits performed in [41] of single-crystal flux-flow resistivities from surface impedance measurements [36], for the H‖c axis and H‖a-b planes, respectively; a value of 0.11 was reported from the field-induced variations of $R_{s}$ in a polycrystalline sample [57]. With the available bibliographic data, considering an averaged orientation for the single crystal values, we took $0.11\leq \sigma_{n,\sigma }/\sigma_{n,\pi }\leq 0.38$[4,41]. Hence, $22MS/s\leq \sigma_{n,\pi }\leq 27.3MS/s$, which we take temperature independent in the temperature range studied. On the other hand, the literature values for λ yielded 85 nm <λ(0)< 180 nm [1]. Thus, we used average values $\sigma_{n,\sigma }/\sigma_{n,\pi }=0.245$, $\sigma_{n,\pi }$ = 24.7 MS/m and λ(0) = 133 nm, observing that letting these quantities vary in the whole range provided in the literature had little (<7%) impact on the accuracies of the vortex parameters in the considered range (*T*, *B*).

**Figure 6:**
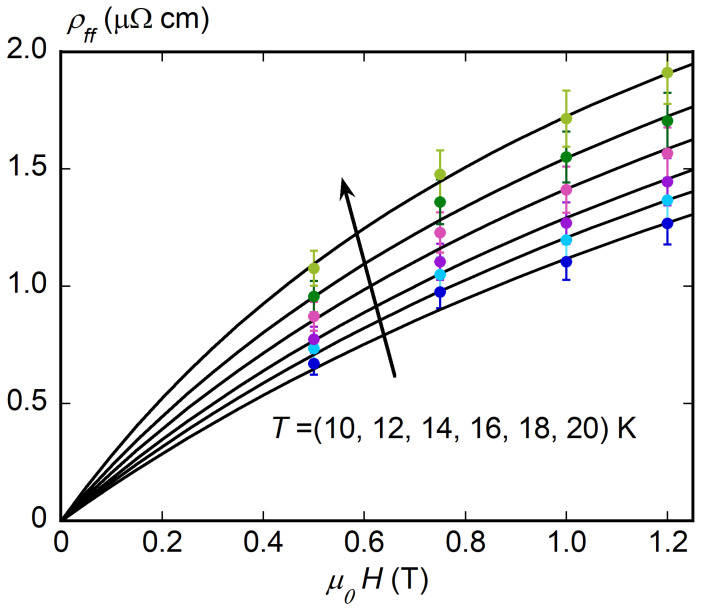
$\rho_{ff}$ vs. H at various *T*, with fits according to Equation (9) reported as dashed lines.

We focused on flux-flow resistivity $\rho_{ff}$, while depinning frequency $\nu_{p}$ and pinning constant $k_{p}$ were extracted and commented in a previous work [55]. The obtained $\nu_{p}$, ≃10 GHz, was deemed to be competitive at low fields with that of the other technological relevant (low-$T_{c}$) superconductor Nb${}_{3}$Sn [58,59]. $\rho_{ff}$ was obtained from the data for $R_{s1}$ and $R_{s2}$, and it is reported against *H* in Figure 6 at different temperatures *T*.

$\rho_{ff}\left(B\right)$ exhibited a downward curvature at all *T* that is a signature of multiband superconductivity [41,60], although not consistently observed in every multigap superconductor, as the results in various iron-based superconductors testify [61,62,63,64,65]. Indeed, assuming the Bardeen–Stephen relation for the flux-flow resistivity [40] of both bands considered individually, given their s-wave pairing symmetry, flux-flow resistivity $\rho_{ff}\left(B\right)$ expression was established in [41] as follows:

(9) $$\begin{array}{c} \rho_{ff}=\frac{H}{\frac{H_{c2}}{\rho_{n,\sigma }}+\frac{H}{\rho_{n,\pi }}}=\frac{1}{\sigma_{n,\pi }}\frac{H}{H+H_{c2}\frac{\sigma_{n,\sigma }}{\sigma_{n,\pi }}}\;, \end{array}$$

where in the last equality, the whole expression was cast in order to render the known quantities explicit (*H*, $\sigma_{n,\pi }$, $\sigma_{n,\sigma }/\sigma_{n,\pi }$). Equation (9) could, thus, be used to fit the experimental data of Figure 6 with $H_{c2}\left(T\right)$ as the only unknown parameter. Fits, reported as continuous lines, reproduce the experimental trend well. Moreover, the fits allowed for extracting $H_{c2}$ at temperatures where a direct measure is beyond the present experimental capability. The corresponding $H_{c2}\left(T\right)$, reported in Figure 3, was consistent with values measured on polycrystalline samples such as the present one [1,53,54] and lay well within the limits set by the anisotropy in $H_{c2}$ as determined by measurements for fields parallel to *a*–*b* planes and the *c* axis performed in single crystals [66,67]. Moreover, they were consistent with the values directly measured at higher temperatures, confirming the consistency of these experimental results with the theoretical model of Equation (9) [41].

## 5. Conclusions

In this paper, we presented measurements of the surface resistance $R_{s}$ in a MgB${}_{2}$ bulk sample fabricated by means of an ex situ spark plasma sintering technique [23]. The measurements were performed with a bitonal dielectric loaded resonator [47,51] tuned at 16.5 and 26.7 GHz in field cooling condition at $\mu_{0}H=\left(0,0.100,0.250,0.375,0.500,0.750,1.000,1.200\right)$ T and for temperatures T>10 K. Two-frequency measurements allowed for data-rooted analysis to establish the relevance of pinning at the frequencies of measurement. In particular, the Coffey–Clem (CC) model [43] for the high-frequency vortex motion resistivity was used to evaluate the flux-flow resistivity $\rho_{ff}$ in this kind of material from the measured $R_{s}$.

The suitability of such a simplified model that does not take into account the complex vortex motion phenomenology typical of multigap superconductors was preliminarily verified. Indeed, the plot of the $R_{s}\left(26.7\;\mathrm{GHz}\right)$ versus $R_{s}\left(16.5\;\mathrm{GHz}\right)$ curves measured at the different *H* fields, and shown in Figure 5, demonstrates how also a single-component treatment of the vortex motion resistivity was able to reproduce the data.

The so-obtained $\rho_{ff}\left(H\right)$ shows the typical negative curvature of multigap superconductors. Thus, taking into account the low interaction of the superconductive bands in MgB${}_{2}$, the field dependence of $\rho_{ff}$ was analyzed with the expression developed by Goryo and Matsukawa [41], and showed good agreement between the model and the experimental data. In addition, the fit of the $\rho_{ff}\left(H,T\right)$ with this model allowed for the determination of the upper critical field, even in the low-*T* region, obtaining field values well beyond those reachable with the used instrumentation.

Lastly, the found values for the upper critical field were compared with the literature data reported for both polycrystalline and single crystal samples [1]. The obtained values were in good agreement with those observed in polycrystalline samples and fell within the range defined by the anisotropy of $H_{c2}$ as obtained on oriented crystals. This allowed for deriving two important conclusions: (i) the intermediate $H_{c2}$ with respect to that of oriented crystals indicates that the anisotropy of the sample was low and in agreement with what is expected with polycrystals; (ii) since the $H_{c2}$ values evaluated here through $\rho_{ff}$ measurements were coherent with the theoretical model [41], no additional evident effects of the granularity of the sample were observable. This last point agrees well with the fact that SPS MgB${}_{2}$ samples have a much higher density (99% in this work) than that of samples fabricated with standard powder metallurgy [23]. Such a high density, close to the theoretical one, combined with quite a low anisotropy factor and high coherence length, ensures that grain boundaries do not have a significant effect on microwave-measured microscopic parameters.

## Data Availability

Data available on request to the corresponding author.
